# Alumni Meeting

**Published:** 1876-04

**Authors:** T. H. Davy

**Affiliations:** Recording Secretary.


### ARTICLE V.

### *Alumni Meeting.*

Pursuant to notice, a meeting of the Graduates of the Baltimore College of Dental Surgery was held on the 6th of March, in the lecture room of the College Building, S. E. Corner of Eutaw and Lexington Sts., with the object of forming an Alumni Association.

About one hundred of the graduates assembled, quite a number coming from other States and cities.

The meeting was organized by the call of Dr. S. J. Cockerille to the chair, Dr. F. J. S. Gorgas acting as Secretary.

The balloting for the officers of the Association for the ensuing year resulted as follows:

Dr. S. J. Cockerille, of Washington, class of '53, President; Dr. T. A. La Far, of Baltimore, class of '60, First Vice-President; Dr. S. D. French, of Troy, New York, class of '54, Second Vice-President; Dr. T. H. Davy, of Baltimore, class of '74, Recording Secretary; Dr. Wm. B. Wise, of Norfolk, Va., class of '74, Corresponding Secretary; Dr. Wm. H. Hoopes, of Baltimore, class of '56, Treasurer. Executive Committee, Drs. F. J. S. Gorgas, Jas. H. Harris and Wm. H. Hoopes.

Dr. Cockerille on assuming the chair, made some interesting remarks on the present strength and dignity of the profession, the necessity for organization, and the claims of their *alma mater* upon those she sends forth to practice the dental art.

A committee, consisting of Drs. J. B. Hodgkin, S. D. French and W. A. Mills, was appointed to draft a Constitution for the Association.

On invitation of the President, Dr. Wm. H. Hoopes addressed the meeting on the subject of his relations with the Goodyear Rubber Company concerning celluloid.

On motion, the members of the present graduating class were elected members of the Association.

A constitution was submitted by the committee above named, and was unanimously adopted. A committee was then appointed to draw up by-laws, consisting of Drs. Gorgas, Wilkerson and Wise, the report of which was also unanimously adopted.

Letters from Drs. W. W. H. Thackston, of Farmville, Va.; Wm. H. Morgan, of Nashville, Tenn.; Samuel Welchens, Lancaster, Pa.; George F. Keesee, Richmond, Va.; Wm. Farmer, of Abingdon, Va.; Dr. Jno. W. Holt, of Goldsboro, N. C.; Dr. F. H. Rehwinkel, of Chillicothe, Ohio, and Dr. N. M. Burkholder, Va., were read, expressing their regrets at not being able to attend, and their wishes for the success of the organization.

On motion, it was resolved to invite Dr. William H. Thackston, of Farmville, Va., to address the Association at its next annual meeting.

The constitution provides that the body be called the "Alumni Association of the Baltimore College of Dental Surgery," and is as follows:

### CONSTITUTION.

---

ARTICLE I.—This Association shall be called "THE ALUMNI ASSOCIATION OF THE BALTIMORE COLLEGE OF DENTAL SURGERY."

ARTICLE II.—The officers of this Association shall consist of a President, one or more Vice-Presidents, a Recording and a Corresponding Secretary, a Treasurer, and an Executive Committee, which shall consist of three members.

The President, Vice-Presidents, Secretary and Treasurer, shall be elected to serve for a period of one year, or until others are elected to fill their places.

The Executive Committee shall be appointed by the President annually.

The duties of the President, Vice-President, Secretary and Treasurer, shall be such as are usually performed by like officers in other organizations.

It shall be the duty of the Executive Committee to provide a place of meeting for the Association; to audit the accounts of the Treasurer, and to perform such other duties as may be required of them by the President.

ARTICLE III.—The object of this Association shall be to encourage and promote the association of dentists with each other; to stimulate free interchange of opinion; to advance the Science of Dental Surgery and to keep alive the love we bear to our *Alma Mater*.

ARTICLE IV.—These laws may be amended at any annual meeting by a two-thirds vote of the members present and voting.

ARTICLE V.—The graduating class of each session, (who have passed examinations), shall be considered as members of this Association.

### BY-LAWS.

---

ARTICLE I.—The regular annual meeting of this Association shall be held on the day of the Annual Commencement of the Baltimore College of Dental Surgery, at 10 o'clock A. M. Special meetings may be called at such other times as the President may deem necessary.

ARTICLE II.—Every active member of this Association shall pay annually the sum of one dollar, as dues to meet current expenses.

Article III.—Any alumnus of the Baltimore College of Dental Surgery may become an active member of this Association on the payment of the annual dues.

T. H. Davy, D. D. S.,

*Recording Secretary.*

